# Clinical Insights and Future Prospects: A Comprehensive Narrative Review on Immunomodulation Induced by Electrochemotherapy

**DOI:** 10.3390/curroncol31100478

**Published:** 2024-10-21

**Authors:** Martina Ferioli, Anna Myriam Perrone, Pierandrea De Iaco, Arina A. Zamfir, Gloria Ravegnini, Milly Buwenge, Bruno Fionda, Erika Galietta, Costanza M. Donati, Luca Tagliaferri, Alessio G. Morganti

**Affiliations:** 1 Radiation Oncology, Azienda Ospedaliero-Universitaria di Ferrara, 44124 Ferrara, Italy; martina.ferioli@ospfe.it; 2 Department of Medical and Surgical Sciences-DIMEC, Alma Mater Studiorum University of Bologna, 40138 Bologna, Italy; myriam.perrone@aosp.bo.it (A.M.P.); pierandrea.deiaco@unibo.it (P.D.I.); mbuwenge@gmail.com (M.B.); erika.galietta2@unibo.it (E.G.); donati.costanza@gmail.com (C.M.D.); alessio.morganti2@unibo.it (A.G.M.); 3 Division of Oncologic Gynecology, IRCCS Azienda Ospedaliero-Universitaria di Bologna, 40138 Bologna, Italy; 4 Radiation Oncology, IRCCS Azienda Ospedaliero-Universitaria di Bologna, 40138 Bologna, Italy; 5 Department of Pharmacy and Biotechnology (FABIT), University of Bologna, 40138 Bologna, Italy; gloria.ravegnini2@unibo.it; 6 UOC di Radioterapia Oncologica, Dipartimento di Diagnostica per Immagini, Radioterapia Oncologica ed Ematologia, Fondazione Policlinico Universitario “A. Gemelli” IRCCS, 00168 Rome, Italy; bruno.fionda@policlinicogemelli.it (B.F.); luca.tagliaferri@policlinicogemelli.it (L.T.)

**Keywords:** electroporation, electrochemotherapy, immunotherapy, immunomodulation, literature review, narrative review

## Abstract

Electrochemotherapy (ECT) is an emerging therapeutic approach gaining growing interest for its potential immunomodulatory effects in cancer treatment. This narrative review systematically examines the current state of knowledge regarding the interplay between ECT and the immune system. Through an analysis of preclinical and clinical studies, the review highlights ECT capacity to induce immunogenic cell death, activate dendritic cells, release tumor antigens, trigger inflammatory responses, and occasionally manifest systemic effects—the abscopal phenomenon. These mechanisms collectively suggest the ECT potential to influence both local tumor control and immune responses. While implications for clinical practice appear promising, warranting the consideration of ECT as a complementary treatment to immunotherapy, the evidence remains preliminary. Consequently, further research is needed to elucidate the underlying mechanisms, optimize treatment protocols, explore potential synergies, and decipher the parameters influencing the abscopal effect. As the field advances, the integration of ECT’s potential immunomodulatory aspects into clinical practice will need careful evaluation and collaboration among clinical practitioners, researchers, and policymakers.

## 1. Introduction

Electrochemotherapy (ECT) is a therapeutic approach that involves the synergistic application of electroporation and chemotherapy for the treatment of cancerous tissues [1].

Electroporation, a biophysical technique that employs short, high-voltage electrical pulses, temporarily disrupts the lipid bilayer of cell membranes, creating transient pores. These pores increase the permeability of the cell membrane, allowing otherwise impermeable molecules, such as chemotherapeutic agents, to enter the cell more efficiently. In the context of cancer treatment, this process is known as ECT, which enhances the intracellular concentration of cytotoxic drugs in tumor cells. The most commonly used drugs in ECT, such as bleomycin or cisplatin, are particularly suited for this technique, as they have poor membrane permeability on their own but exhibit potent cytotoxicity once inside the cell [1,2,3].

The process begins with the administration of the chemotherapeutic agent, either intravenously or intratumorally, followed by the application of electrical pulses directly to the tumor site. These pulses are carefully controlled in terms of duration, amplitude, and frequency to ensure optimal permeabilization of the tumor cells without causing excessive damage to surrounding healthy tissue. Once the electric field is applied, the temporarily permeabilized cells allow the chemotherapeutic drugs to enter at significantly higher concentrations than would occur without electroporation.

ECT efficacy stems from its dual mechanism of action. First, the electroporation process increases the intracellular uptake of the chemotherapeutic agents, enhancing their cytotoxic potential. Second, the drugs themselves exert their antineoplastic effects, such as causing DNA strand breaks (in the case of bleomycin) or forming DNA adducts that inhibit DNA repair (as seen with cisplatin). Together, these mechanisms lead to enhanced tumor cell death.

ECT has been primarily used in the treatment of various cutaneous and subcutaneous tumors, including melanoma and breast cancer [1,4], particularly for skin metastases, basal [5] and squamous cell carcinoma [6], vulvar carcinoma [7,8], and other skin and soft tissue tumors such as cutaneous lymphomas [9], sarcomas [10], and Kaposi’s sarcoma [11].

In fact, the ECT primary applications are in tumors that are accessible and superficial, where the localized cytotoxicity of the technique and tumor cell death induction can be effectively harnessed. However, research continues to explore its potential application in deeper-seated tumors [12,13,14].

ECT has the theoretical potential to exert immunomodulatory activity through several mechanisms [15]. In fact, ECT has the potential to exert immunomodulatory activity through several mechanisms. The innate immune response is first triggered when dying tumor cells release damage-associated molecular patterns, such as intracellular adenosine triphosphate (ATP) and High Mobility Group Box 1 (HMGB1) protein. These molecules attract immune cells, including macrophages and neutrophils, to the site of tumor destruction, promoting an inflammatory response.

ECT also induces the activation of dendritic cells (DCs), key antigen-presenting cells that play a crucial role in bridging innate and adaptive immunity. Activated DCs process tumor antigens and present them to T cells, initiating an adaptive immune response against the tumor. As tumor cells are destroyed, antigens are released and can be taken up by antigen-presenting cells like DCs, further supporting the development of a specific antitumor immune response.

**Figure 1:**
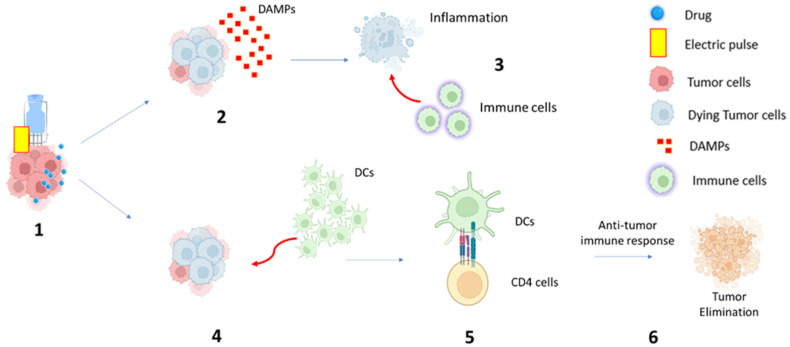
Hypothetical mechanism of electrochemotherapy-induced immune response: (**1**) Electric pulse is applied to the tumor mass, and the chemotherapy drug can be delivered within the tumor cell, amplifying the cytotoxic effect; (**2**) Dying tumor cells release signals that alert the immune system, including damage-associated molecular patterns (DAMP) release; (**3**) DAMPs attract immune cells to the site of tumor destruction and promote an inflammatory response; (**4**) At the same time, ECT can lead to the recruitment and activation of dendritic cells (DCs); (**5**) Activated DCs process tumor antigens and present them to T cells; (**6**) Antitumor immune response and tumor elimination.

Additionally, ECT can lead to the local release of cytokines and chemokines, contributing to immune cell recruitment and the generation of a broader immune response. This local immune response may occasionally manifest in systemic immune activity, known as the abscopal effect, where distant, untreated tumor sites are affected by immune activation [16] (Figure 1).

While ECT itself is not a replacement for other immunotherapies, its immunomodulatory activity can complement other approaches, potentially enhancing the overall efficacy of the treatment.

To address gaps in our current understanding and to provide a consolidated overview of the evolving landscape surrounding ECT-induced immunomodulation, this narrative review aims to critically assess the existing evidence, offer insights into potential mechanisms, and discuss the implications for clinical practice and future research.

## 2. Material and Methods

### 2.1. Literature Search Strategy

The review was registered on the PROSPERO database before literature screening (registration number CRD42021239102) [17]. A comprehensive narrative review of the existing literature on the immunomodulation induced by ECT was carried out [Table S1]. The review included English-language papers without any time limitations. The snowball technique, along with systematic database searches, was employed to identify both primary articles and relevant references. In particular, we used both the ‘backward’ and ‘forward’ snowballing methods [1]. The databases PubMed, Scopus, and Cochrane Library were systematically searched for relevant articles. The search strategy included various combinations of keywords, such as “electroporation”, “electrochemotherapy”, “immunotherapy”, “immune system”, and “immune-stimulation”.

### 2.2. Inclusion and Exclusion Criteria

Papers were included if they focused on the immunomodulatory effects of ECT on tumor microenvironments or immune responses. Studies could include both preclinical and clinical investigations. Abstracts, editorials, reviews, and commentaries were excluded to ensure a focus on original research articles. Furthermore, studies reporting the results of combined modality treatments based on the association of ECT and immunotherapy were excluded.

### 2.3. Data Extraction and Synthesis

A structured approach was used to extract relevant data from the selected articles. Key information, including study design, tumor types, treatment protocols, immunomodulatory mechanisms investigated, and reported outcomes, was extracted. Data were synthesized to provide a coherent overview of the current understanding of how ECT influences immune responses.

### 2.4. Study Selection

The initial database search yielded a total of 236 articles. After removing duplicates, the titles and abstracts of the remaining articles were screened for relevance. Full texts of potentially relevant articles were then assessed based on the inclusion and exclusion criteria. The final number of articles included in the review was nine.

## 3. Results

In total, we identified 14 studies to be included in the present narrative review. Of them, 12 were preclinical studies on mice [16,18,19,20,21,22,23,24,25,26,27,28], and 2 were clinical studies, both including patients with melanoma skin metastases [29,30]. Characteristics and results of the studies are summarized in Table 1 (preclinical studies) and Table 2 (clinical studies).

### 3.1. Preclinical Studies

Sersa et al. conducted an in vitro study using fibrosarcoma SA-1 tumors in mice. The aim was to determine the effects of ECT on natural resistance and immune responsiveness. ECT was administered with bleomycin and involved using two flat parallel stainless-steel electrodes. The results showed a 52% complete response rate in tumor elimination. Phagocytic activity remained unchanged, but T lymphocyte activity increased 14 days after ECT. This indicated that ECT enhanced monocyte oxidative burst and T lymphocyte activity. The study concluded that ECT could improve the immune response by enhancing monocyte function [18].

Another study by Sersa et al. investigated the adjuvant effect of TNF-α on ECT antitumor effectiveness in the same animal model. ECT with bleomycin was combined with TNF-alfa using two flat, parallel stainless-steel electrodes. While TNF-α had some antitumor effect, the combination of ECT and TNF-α showed a significant increase in the median survival times of mice. The study suggested that TNF-α might augment the antitumor activity of ECT through immunomodulation [19].

Sersa et al. [24] also conducted a study comparing the antitumor effectiveness of ECT with cisplatin in immunocompetent and immunodeficient mice with LPB sarcoma. The tumor growth delay in immunocompetent mice was about twice as long as in immunodeficient mice, and tumor cures were only achieved in immunocompetent mice. These results emphasize the crucial role of the immune system in the success of ECT with cisplatin [23].

Roux et al. [20] explored the release of tumor-associated antigens and systemic immunity after ECT combined with CpG ODN (an immunoadjuvant) in a sarcoma model in mice. ECT induced recruitment of immune cells and an increase in TLR9 expression. The combination of ECT and CpG ODN led to complete regression in local tumors and significant rejection of distant, untreated tumors, showcasing potent local and systemic antitumor responses. The study highlighted the synergistic effects of ECT and immunoadjuvants in inducing immune responses [20].

Sedlar et al. [25] explored the effects of combining ECT with intramuscular interleukin-12 (IL-12) gene electrotransfer in murine sarcoma and carcinoma models. ECT was more effective in the more immunogenic sarcoma model, with 17% tumor cures. IL-12 gene electrotransfer further increased the tumor response, particularly in the sarcoma model, emphasizing the importance of tumor immunogenicity in treatment effectiveness” [25].

The work by Calvet et al. focused on immunogenic cell death induced by ECT in murine colon cancer cells. ECT-treated cells released ATP and HMGB1, signaling immunogenicity. Immunocompetent mice vaccinated with ECT-treated cells exhibited a high complete response rate and protection against subsequent challenges. The study concluded that ECT generated immunogenic cell death (ICD), partly due to electroporation, promoting antitumor immunity [21].

Tremble et al. [22] investigated the combination of ECT with an activating inducible T cell costimulator (ICOS) antibody in lung carcinoma and colorectal cancer cells in mice. ECT combined with ICOS activation led to increased infiltration of immune cells, decreased tumor volume, and improved overall survival. This combination showed strong curative outcomes without significant autoimmunity risk [22].

Subsequently, Ursic et al. [23] compared ECT with oxaliplatin or cisplatin in murine melanoma cells. ECT with both agents induced significant lymphocyte infiltration and delayed tumor growth, with cisplatin exhibiting more prolonged effects. ECT with oxaliplatin and cisplatin promoted immunological components in the antitumor response, indicating their potential as immune-stimulating treatments [23].

In another study by Tremble et al. [22], ECT combined with cisplatin was investigated in multiple tumor models. ECT significantly enhanced immune cell recruitment, reduced tumor growth, and exerted an abscopal effect by reducing growth in distant, untreated tumors. This study highlighted the ECT potential to modulate tumor microenvironments and induce systemic immune responses [16].

Polajzer et al. [26] studied the release of damage-associated molecular patterns (DAMPs) following electroporation in vitro. Using hamster ovary cells, the study found that higher pulse amplitudes led to increased DAMP release, which correlated with cell death. These findings suggest that DAMPs could serve as markers to predict cell death and immune activation after electroporation [26].

Ursic et al. [27] investigated the combination of ECT with IL-12 gene electrotransfer in murine tumor models with varying immunogenicity. In poorly immunogenic melanoma, IL-12 gene electrotransfer potentiated the effects of ECT, leading to a complete response in 38% of cases and inducing an abscopal effect. However, more immunogenic tumors, like 4T1 mammary carcinoma and CT26 colorectal carcinoma, responded better to ECT alone, indicating that the effectiveness of combination therapy depends on tumor immune status [27].

Kesar et al. [28] examined the immunogenic effects of ECT in murine tumor cell lines, analyzing the release of DAMPs and the expression of immune markers. The study found that ECT induced immunogenic cell death (ICD) and altered the expression of immune markers, though the effects were tumor-type and chemotherapeutic agent-specific [28].

### 3.2. Clinical Studies

The first clinical study was presented by Gerlini et al. [29], who investigated dendritic cell presence after ECT in melanoma skin metastases in nine patients. ECT promoted the migration of dendritic cells from tumors to lymph nodes and recruited plasmacytoid dendritic cells (pDCs) and conventional dendritic cells (dDCs) to the lesion site. This highlighted the potential for combining ECT with in situ dendritic cell activation for a new therapeutic approach in metastatic melanoma patients [29].

In a clinical study, Di Gennaro et al. [30] explored the effects of ECT on T cell subsets in 10 patients with melanoma skin metastases. ECT reduced CD4-FOXP3 Treg cells and increased CD3-CD8 T cell frequency in the perilesional dermis. Despite local immune presence, ECT alone did not induce complete immune responses, implying the need for additional strategies to enhance systemic immunity [30].

## 4. Discussion

ECT has been suggested to potentially elicit immunomodulatory effects through postulated mechanisms, including but not limited to immunogenic cell death, dendritic cell activation, tumor antigen release, inflammatory responses, local cytokine release, and, in some instances, the conjectured induction of systemic effects, commonly known as the abscopal effect. These assumed combined mechanisms have led to the hypothesis that ECT could establish a conducive microenvironment for immune cell activation and recruitment, thus theoretically promoting an antitumor immune response. However, it should be noted that while these hypotheses propose a role for ECT in modulating the immune system, ECT is not positioned as a replacement for existing immunotherapies. Instead, it is speculated that the potential immunomodulatory properties of ECT might complement these established approaches, presenting an avenue for potentially enhancing the efficacy of cancer treatment strategies.

ECT has demonstrated immunomodulatory effects across multiple preclinical and clinical studies, highlighting its capacity to activate both local and systemic immune responses. Preclinical studies consistently show that ECT can trigger immunogenic cell death (ICD) through the release of damage-associated molecular patterns (DAMPs), such as ATP and HMGB1, which play a key role in immune activation. For example, Calvet et al. [21] demonstrated that ECT induced ICD markers and promoted an effective antitumor immune response, particularly when combined with bleomycin. Similarly, Polajzer et al. [26] showed that DAMP release increased with higher pulse amplitudes and was closely linked to cell death, further supporting ECT’s role in initiating immune activation. Studies like those of Sersa et al. [18,24] emphasized the importance of the immune system in enhancing the antitumor effectiveness of ECT, with stronger responses seen in immunocompetent mice compared to immunodeficient ones. The addition of immunomodulatory agents, such as TNF-α and IL-12, was shown to further augment these effects by increasing tumor cell kill rates and systemic immune responses (Sersa et al. [19]; Sedlar et al. [25]).

In addition to enhancing local immune responses, several studies have demonstrated ECT’s potential to generate systemic effects. Roux et al. [20] showed that combining ECT with CpG oligodeoxynucleotides led to both local tumor regression and rejection of distant, untreated tumors, suggesting that ECT can induce systemic T-cell-mediated responses. Similar results were observed in Tremble et al. [16], where ECT combined with cisplatin mobilized immune cells and showed abscopal effects, reducing growth in untreated tumors. These findings were further supported by studies that combined ECT with IL-12 gene electrotransfer, demonstrating that IL-12 could potentiate the systemic immune effects of ECT, particularly in less immunogenic tumor models (Ursic et al. [27]).

Clinical studies have echoed these immunomodulatory effects, as seen in Gerlini et al. [29], where ECT induced dendritic cell recruitment and activation in melanoma metastases, highlighting its potential for in situ DC vaccination strategies. Moreover, Di Gennaro et al. [30] demonstrated that ECT in melanoma patients reduced regulatory T cells (Tregs) and increased CD8+ T cells, indicating that ECT promotes a favorable immune environment for tumor clearance and could be effectively combined with immunotherapies to achieve durable responses. Together, these studies underscore the importance of the immune system in determining the success of ECT and point to the potential of combining ECT with immunomodulatory agents to further enhance both local and systemic antitumor effects.

However, there are several limitations and areas for further investigation that should be considered. In fact, while preclinical studies have provided valuable insights into ECT mechanisms and effects, translating these findings to clinical settings can be complex. Factors such as tumor microenvironment differences between animal models and human tumors, as well as potential variations in immune responses, need to be carefully addressed. Additionally, while some clinical studies have been carried out [23,24], the number of patients and trials remains relatively limited. Larger trials are needed to establish the safety, efficacy, and optimal protocols for ECT in different tumor types. Furthermore, the observed response to ECT can vary among different tumor types and patients. Further research is needed to understand the factors influencing these variations, including tumor size, location, and intrinsic characteristics.

Some studies have explored the combination of ECT with immunomodulatory agents, but the optimal combinations, doses, and sequencing are yet to be fully elucidated. Identifying synergistic therapies that enhance the immunomodulatory effects of ECT could improve overall treatment outcomes. Moreover, while ECT has demonstrated immunomodulatory effects, the specific mechanisms underlying these responses require further investigation. Understanding the interactions between ECT-induced cell death, immune cell recruitment, and systemic immune activation is essential. Furthermore, the long-term effects of ECT on the immune system, including the potential development of immunological memory against the treated tumor, remain areas of ongoing study.

Additionally, the heterogeneity in ECT protocols (electrode types, voltage, number of pulses, drug combinations) across different studies makes it challenging to establish standardized guidelines. Developing consistent protocols will aid in comparing results across studies and optimizing treatment outcomes. Finally, while ECT is primarily a locoregional therapy, investigating its combination with systemic immunotherapies could enhance its impact by triggering systemic antitumor immune responses, and further research is needed to understand the mechanisms underlying the abscopal effect observed in some studies, where ECT-treated tumors impact distant, untreated tumors.

## 5. Conclusions

In conclusion, ECT demonstrates significant immunomodulatory effects that could complement current cancer therapies. While preclinical and early clinical studies have highlighted ECT’s potential to induce immunogenic cell death, activate immune cells, and trigger systemic immune responses, including the abscopal effect, recent research remains limited in terms of large-scale clinical validation. The promising results observed in smaller studies underscore the need for well-designed, large-scale clinical trials to better understand the mechanisms underlying ECT effects, optimize treatment protocols, and explore combinations with immunotherapy and other systemic treatments [31].

Future research should focus on standardizing ECT protocols and evaluating their long-term efficacy across diverse tumor types and patient populations. Despite gaps in the current body of research, the integration of ECT into multimodal cancer treatment regimens represents a valuable avenue for enhancing therapeutic outcomes. For health professionals, these findings highlight the multifaceted nature of ECT’s impact on tumor microenvironments. Incorporating ECT into clinical practice could offer an innovative approach that not only directly targets tumor cells but also harnesses the immune system power for enhanced therapeutic outcomes. Health practitioners should consider the potential for immunomodulation when designing treatment regimens, potentially coupling ECT with other immunotherapeutic strategies to optimize patient outcomes.

From a policy development and implementation perspective, the review emphasizes the need for increased awareness and education among healthcare providers about ECT immunomodulatory potential. Policies should reflect the evolving landscape of cancer treatment, acknowledging the role of ECT as a bridge between local therapy and immune-based interventions. Integrating ECT into treatment guidelines could ensure that patients receive well-rounded care, addressing not only the primary tumor but also its potential metastatic spread and systemic immune response.

## Figures and Tables

**Table 1:** Preclinical studies.

Authors, Year	Aims	Methods	Results	Conclusions
Sersa et al., 1996 [18]	Determine the effects of ECT with bleomycin on immune responsiveness.	ECT applied to SA-1 tumors in mice evaluated phagocytic activity, blast transformation of spleen cells, and oxidative burst.	Increased oxidative burst in monocytes and higher T-lymphocyte activity 7–14 days post-treatment.	ECT affects immune system function, enhancing natural resistance and immune responsiveness.
Sersa et al., 1997 [19]	Increase the antitumor effectiveness of ECT by adding TNF-α.	Combination of ECT with bleomycin and TNF-α in SA-1 tumors in mice, administered intratumorally or peritumorally.	Enhanced antitumor effectiveness when combined with TNF-α; independent of interaction with bleomycin or electric pulses.	TNF-α has an immunomodulatory effect, improving the antitumor activity of ECT and adding a systemic component.
Sersa et al., 1997 [24]	Compare ECT with cisplatin in immunocompetent and immunodeficient mice.	ECT applied to LPB sarcoma in immunocompetent and immunodeficient mice assessed tumor growth delay and cure rates.	Tumor growth delay was twice as long in immunocompetent mice, and a higher tumor cure rate was achieved compared to immunodeficient mice.	The immune system is critical for the success of ECT with cisplatin, indicating the importance of immune responsiveness.
Roux et al., 2008 [20]	Investigate whether ECT can induce systemic immunity when combined with CpG.	ECT combined with CpG oligodeoxynucleotide injections in murine models analyzed tumor responses, cellular recruitment, and TLR9 expression.	Induced systemic T-dependent antitumor response and triggered tumor-specific T-cell effectors, leading to both local and distant antitumor effects.	ECT combined with CpG enhances both local and systemic antitumor effects, suggesting a potential vaccination strategy.
Sedlar et al., 2012 [25]	Test the combination of ECT with cisplatin and IL-12 gene electrotransfer.	ECT with cisplatin combined with IL-12 gene electrotransfer in sarcoma and carcinoma models, evaluated in vitro and in vivo for immune and tumor responses.	Combined therapy increased tumor cell kill, growth delay, and cure rates, especially in more immunogenic sarcomas, with an IL-12 dose-dependent response.	IL-12 gene electrotransfer enhances the effectiveness of ECT, particularly in more immunogenic tumors, emphasizing the importance of immune competence.
Calvet et al., 2014 [21]	Evaluate ECT’s ability to induce ICD in cancer cells.	In vitro analysis of CT26 murine colon cancer cells treated with ECT and electric pulses was assayed for immunostimulatory markers (CRT, ATP, HMGB1).	ECT-induced ICD markers include CRT exposure and ATP release. Combination with bleomycin is necessary for HMGB1 release and an effective antitumor immune response.	ECT not only causes direct cytotoxicity but also induces a systemic anticancer immune response via ICD activation.
Tremble et al., 2018 [22]	Examine the effect of ICOS activation with ECT.	Murine models of primary and metastatic tumors treated with ECT and ICOS activation.	Combined therapy led to effective tumor clearance, especially in primary tumors, and survival after tumor rechallenges with immunological memory.	The combination of ECT with ICOS activation enhances immunological responses, promoting local and distant tumor clearance.
Ursic et al., 2018 [23]	Compare the effectiveness of oxaliplatin and cisplatin in ECT for melanoma.	ECT was applied to murine B16F10 melanoma using oxaliplatin and cisplatin, analyzing immunogenic cell death and immune cell infiltration.	Both oxaliplatin and cisplatin induced comparable immunogenic cell death and lymphocyte infiltration, with oxaliplatin requiring a higher dose for equal effectiveness.	Oxaliplatin is as effective as cisplatin in ECT, both inducing similar immune responses.
Tremble et al., 2019 [16]	Explore the efficacy of ECT with cisplatin in murine lung and colorectal cancer models.	ECT with cisplatin applied to lung and colorectal cancer models assessed tumor growth, metastatic potential, and immune cell recruitment.	Cisplatin-based ECT reduced tumor growth and metastatic potential, mobilized immune cells, and showed abscopal effects on untreated tumors.	Cisplatin is effective in ECT for reducing tumor burden and inducing an immune response, with the potential for abscopal effects.
Polajzer et al., 2020 [26]	Analyze DAMP release after electroporation of cells.	Electroporation applied to hamster ovary cells measured DAMP (ATP, calreticulin, and nucleic acids) release and correlated with cell survival.	DAMP release correlated with cell death; increased release was observed with higher pulse amplitudes and time intervals.	DAMP release serves as a marker for predicting cell death and immune activation following electroporation.
Ursic et al., 2021 [27]	Investigate IL-12’s potentiation of ECT effectiveness based on tumor immune status.	ECT with cisplatin, oxaliplatin, or bleomycin combined with IL-12 gene electrotransfer in different murine tumor models.	IL-12 enhanced ECT’s effectiveness in poorly immunogenic tumors but had less impact on more immunogenic tumors, with the greatest effect observed with cisplatin.	The effectiveness of combination therapy depends on tumor immune status, suggesting tailored approaches for better therapeutic outcomes.
Kesar et al., 2023 [28]	Evaluate immunogenic modifications in tumor cells induced by ECT.	ECT applied to murine tumor cell lines (B16-F10, 4T1, CT26) measured DAMP release and expression of immune markers (MHC I, II, PD-L1, CD40).	ECT induced ICD-related DAMPs and immune marker changes in a cell line- and drug-specific manner, demonstrating varying effects across tumor types and drugs.	ECT induces immunogenic modifications in tumor cells, highlighting its potential to enhance antitumor immunity, which varies by tumor type and chemotherapeutic agent.

Legend: DAMP: damage-associated molecular patterns; ECT: electrochemotherapy; ICD: immunogenic cell death; ICOS: Inducible Co-Stimulators.

**Table 2:** Clinical studies.

Authors/Year	Aims	Methods	Results	Conclusions
Gerlini et al., 2013 [29]	Investigate whether ECT can induce antitumor immunity by examining DC recruitment in melanoma metastases.	Biopsies from 9 melanoma patients were taken before and after ECT, followed by immunofluorescence analysis to study DC-related markers in the inflammatory infiltrate.	ECT caused a significant reduction in LCs at day 7, followed by their replacement at day 14. Plasmacytoid and dermal DCs increased significantly after ECT, with some showing activation markers.	ECT promotes migration of LCs to lymph nodes and recruits plasmacytoid and dermal DCs to the lesion site, suggesting potential for in situ DC vaccination strategies.
Di Gennaro et al., 2016 [30]	Examine the effect of ECT on T cell subsets in melanoma metastases, focusing on Tregs and CD8+ T cells.	Fluorescent immunohistochemistry was performed on biopsies from 10 melanoma patients before and after ECT to analyze T cell subsets and markers of immunogenic cell death.	ECT led to a significant decrease in Tregs and an increase in CD8+ T cells at day 14, along with the presence of calreticulin, indicating immunogenic cell death in metastatic cells.	ECT induces local immune responses, characterized by reduced Tregs and increased CD8+ T cell recruitment, suggesting potential for combining ECT with immunotherapy for long-lasting antitumor immunity.

Legend: DC: dendritic cell; ECT: electrochemotherapy; LC: Langerhans cell; Tregs: regulatory T cells.

## Data Availability

Data supporting the reported results will be made available upon reasonable request.

## Supplementary Materials

The following supporting information can be downloaded at: https://www.mdpi.com/article/10.3390/curroncol31100478/s1, Table S1: Narrative review checklist.
