# Supplementary material for: Clinical Insights and Future Prospects: A Comprehensive Narrative Review on Immunomodulation Induced by Electrochemotherapy
Source: Curr Oncol. 2024 Oct 21;31(10):6433–44. doi: 10.3390/curroncol31100478 (PMC11506219; doi:10.3390/curroncol31100478)
Supplement: Supplementary file 1 [file curroncol-31-00478-s001.zip › curroncol-3174052-supplementary.pdf]

Supplementary Material

Table S1: Narrative review checklist.

Section/topic	#	Checklist item	Reported on page or line #
**TITLE**			
title	1	Identify the report as a Narrative Review	Page 1
**ABSTRACT**			
Unstructured summary	2	Provide an unstructured summary including, as applicable: background. Objective, brief summary of narrative review and implications for future research, and clinical practice or policy development.	Page 3
**INTRODUCTION**			
Rationale/background	3	Describe the rationale for the review in the context of what is already known	Page 4
Objectives	4	Specify the key question(s) for the review topic	Page 5
**METHODS**			
Research selection	5	Specify the process for identifying the literature search (e.g. years considered, language, publication status, study design, and databases of coverage	Page 5
**DISCUSSION/SUMMARY**			
Narrative	6	Discuss: 1) research reviewed including fundamental or key findings, 2) limitations and/or quality of research reviewed, and 3) need for future research.	Page 10
Summary	7	Provide and overall interpretation of the narrative review in the context of clinical practice for health professionals, policy development and implementation, or future research.	Page 11
